# Human Cytomegalovirus UL138 Protein Inhibits the STING Pathway and Reduces Interferon Beta mRNA Accumulation during Lytic and Latent Infections

**DOI:** 10.1128/mBio.02267-21

**Published:** 2021-12-14

**Authors:** Emily R. Albright, Clayton K. Mickelson, Robert F. Kalejta

**Affiliations:** a Institute for Molecular Virology, University of Wisconsin—Madison, Madison, Wisconsin, USA; b McArdle Laboratory for Cancer Research, University of Wisconsin—Madison, Madison, Wisconsin, USA; St. Jude Children’s Research Hospital

**Keywords:** herpes, HCMV, persistent, latency, productive, innate immunity, PRR, PAMP, cGAS, TBK1, latent

## Abstract

The cGAS/STING/TBK1 (cyclic guanine monophosphate-AMP synthase/stimulator of interferon genes/Tank-binding kinase 1) innate immunity pathway is activated during human cytomegalovirus (HCMV) productive (lytic) replication in fully differentiated cells and during latency within incompletely differentiated myeloid cells. While multiple lytic-phase HCMV proteins neutralize steps along this pathway, none of them are expressed during latency. Here, we show that the latency-associated protein UL138 inhibits the cGAS/STING/TBK1 innate immunity pathway during transfections and infections, in fully differentiated cells and incompletely differentiated myeloid cells, and with loss of function and restoration of function approaches. UL138 inhibits the pathway downstream of STING but upstream of interferon regulatory factor 3 (IRF3) phosphorylation and NF-κB function and reduces the accumulation of interferon beta mRNA during both lytic and latent infections.

## INTRODUCTION

The innate immune system consists of pattern recognition receptors (PRRs) that detect pathogen-associated molecular patterns (PAMPs) and initiate signal transduction cascades that ultimately lead to the production of interferons (IFNs) and the interferon-stimulated genes (ISGs) that combat and control bacterial and viral infections (1). The double-stranded DNA (dsDNA) genomes of herpesviruses such as human cytomegalovirus (HCMV) are PAMPs detected by the innate immune sensor cyclic guanine monophosphate-AMP synthase (cGAS) (2–4). When cGAS binds to double-stranded DNA, it synthesizes the small molecule cyclic guanine monophosphate-AMP (cGAMP), which, in turn, binds to and activates the stimulator of interferon genes (STING). STING then migrates from the endoplasmic reticulum (ER) to the Golgi apparatus, where it associates with and activates Tank-binding kinase 1 (TBK1). TBK1 directly phosphorylates and thus activates interferon regulatory factor 3 (IRF3). TBK1 also leads to the phosphorylation of the inhibitor of nuclear factor kappa light chain enhancer of activated B cells (iKB), thereby activating nuclear factor kappa light chain enhancer of activated B cells (NF-κB). IRF3 and NF-κB translocate to the nucleus, where they transactivate the transcription of interferon beta (IFN-β) (5–7).

HCMV productively infects highly differentiated cells, such as fibroblasts, macrophages, and epithelial, endothelial, smooth muscle, and dendritic cells (8, 9). The cGAS/STING/TBK1 pathway has been shown to mediate an IFN response to HCMV infection in fibroblasts (10–14), endothelial cells (15), macrophages (12, 16), and dendritic cells (12, 16). Many viruses encode one or more proteins that inactivate innate immunity pathways (17), including the cGAS/STING/TBK1 pathway. Indeed, HCMV encodes 9 proteins known to inactivate the cGAS/STING/TBK1 pathway that are among the ∼200 proteins expressed during productive infections of highly differentiated cells: UL31 (13), UL35 (11), UL42 (12), UL48 (18), UL82 (pp71) (19, 20), UL83 (pp65) (21), UL94 (22), UL122 (IE2) (14), and Us9 (10).

HCMV also latently infects incompletely differentiated cells of the myeloid lineage, such as CD34^+^ hematopoietic progenitor cells (HPCs) and monocytes (23, 24). HCMV-infected THP-1 monocytes show higher IFN-β secretion and increased accumulation of phosphorylated IRF3 than do THP-1 derivatives lacking cGAS or STING (16), indicating that the cGAS/STING/TBK1 pathway also contributes to the IFN response to latent HCMV infection within an incompletely differentiated myeloid cell type. During latency within incompletely differentiated myeloid cells, the productive-phase proteins listed above that inhibit the cGAS/STING/TBK1 pathway do not accumulate, although a different subset of HCMV proteins do accumulate and act during latency (25, 26). To date, no HCMV protein that accumulates within latently infected incompletely differentiated myeloid cells has been demonstrated to inhibit the cGAS/STING/TBK1 pathway.

Here, we show that the HCMV UL138 protein, which is expressed during latency (27), inactivates the cGAS/STING/TBK1 pathway and during infection reduces the accumulation of the mRNA for IFN-β in both highly differentiated fibroblasts permissive for HCMV productive replication and incompletely differentiated myeloid cells (THP-1 monocytes and primary CD34^+^ HPCs) that support HCMV latency. UL138 localizes to the Golgi apparatus (28), a known platform for innate immunity (29), but has not previously been implicated in innate immune evasion. Our work adds to the list of HCMV factors that inactivate the cGAS/STING/TBK1 pathway during productive infections of highly differentiated cells and initiates the list of those that do so during latency within incompletely differentiated myeloid cells.

## RESULTS

### HCMV UL138 colocalizes and interacts with STING.

**FIG 1:**
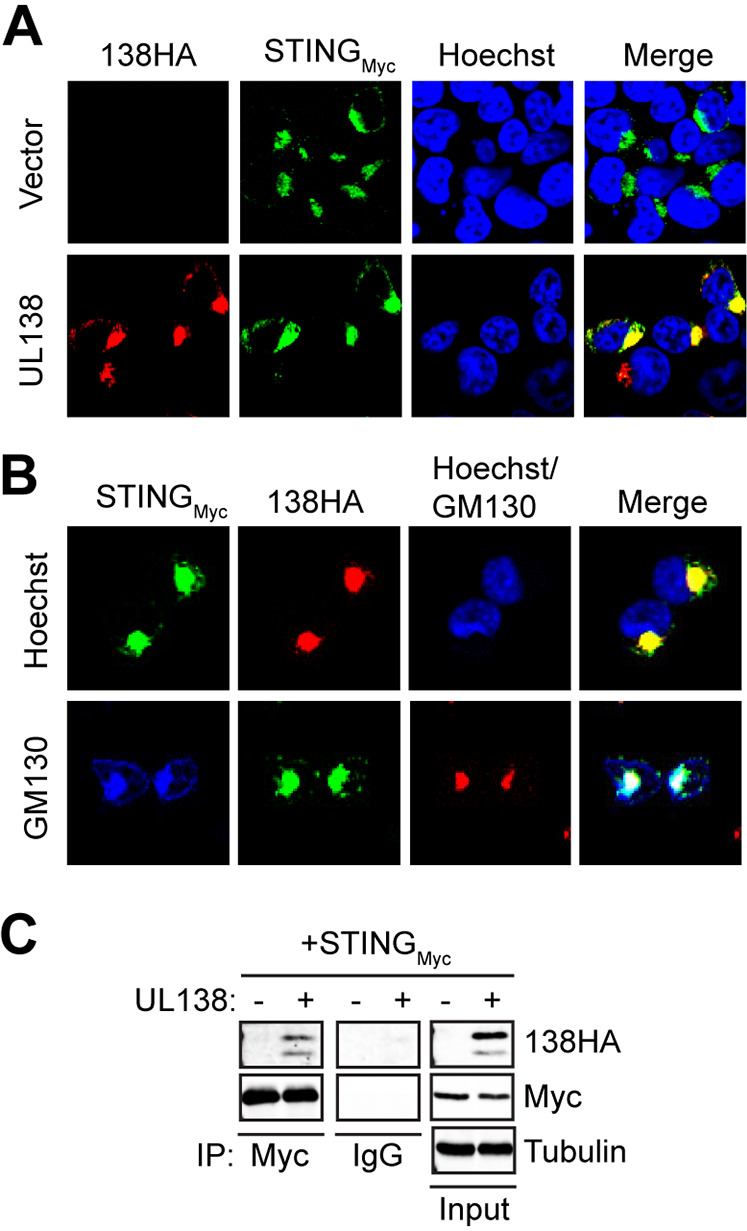
HCMV UL138 colocalizes and interacts with STING. (A) 293T cells cotransfected with expression constructs for cGAS plus STING and either empty vector or UL138 for 48 h were stained for HA-tagged UL138 and Myc-tagged STING. Nuclei were counterstained with Hoechst (*n* = 3). (B) 293T cells cotransfected as for panel A were stained for HA-tagged UL138, Myc-tagged STING, and Golgi marker GM130 (lower images). In upper images, nuclei were counterstained with Hoechst (*n* = 3). (C) Coimmunoprecipitation experiments with 293T cells transfected with expression constructs for cGAS plus Myc-tagged STING and either empty vector (-) or HA-tagged UL138 (+) for 48 h, with Western blotting performed for the indicated proteins (*n* = 3).

An HCMV-encoded STING antagonist expressed during latency has not yet been identified. Based on the localization of the UL138 at the Golgi apparatus, where STING signaling complexes form, we tested whether the UL138 latency protein colocalized or interacted with STING. It is standard to initiate examinations of the STING pathway in HEK293T (293T) cells that do not express endogenous cGAS or STING (30) but fully activate the pathway when these proteins are provided by transient transfection (30–33). In cotransfected 293T cells, UL138 colocalized (Fig. 1A) with STING at the Golgi apparatus, as evidenced by both proteins colocalizing with the Golgi marker GM130 (Fig. 1B) in indirect immunofluorescence experiments. UL138 also interacted with STING in coimmunoprecipitation experiments (Fig. 1C). We conclude that UL138 colocalizes and interacts with STING.

### HCMV UL138 inhibits cGAS/STING-mediated induction of the IFN-β promoter.

**FIG 2:**
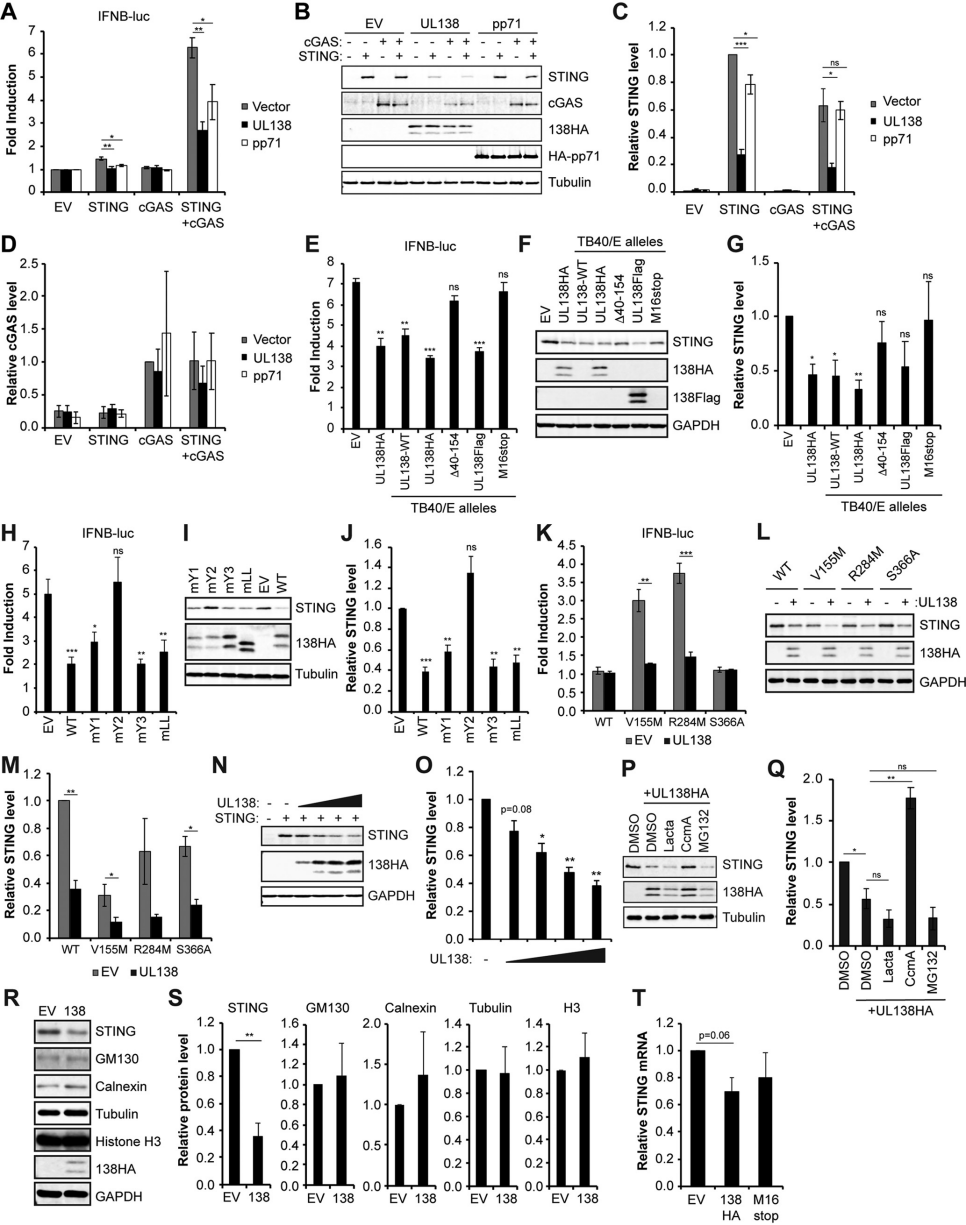
HCMV UL138 inhibits cGAS/STING-mediated induction of the interferon beta promoter and decreases STING steady-state levels via the lysosome. (A) 293T cells cotransfected with IFN-β promoter-driven firefly luciferase construct along with either empty vector (EV) or expression constructs for the indicated protein(s) for 48 h. Fold induction of IFN-β promoter luciferase activity relative to the control with no cGAS or STING is shown (*n* = 4). (B) Representative Western blot for the indicated proteins from lysates from panel A. Tubulin served as a loading control (*n* = 4). (C) Quantitation of STING protein levels from panel B normalized to tubulin levels and shown relative to STING-only transfected controls from the same blot (*n* = 4). (D) Quantitation of cGAS protein levels from panel B normalized to tubulin levels and shown relative to cGAS-only transfected controls from the same blot (*n* = 4). (E) IFN-β promoter luciferase reporter assays as in panel A from 293T cells cotransfected with cGAS plus STING and either EV or the indicated UL138 expression construct made from recombinant TB40/E viruses. Fold induction of IFN-β promoter luciferase activity relative to the control with no cGAS or STING is shown (*n* = 3). (F) Representative Western blot for the indicated proteins from lysates from panel E. GAPDH served as a loading control (*n* = 3). (G) Quantitation of STING levels from panel F normalized to GAPDH levels and shown relative to EV control from the same blot (*n* = 3). (H) IFN-β promoter luciferase reporter assays as in panel A from 293T cells cotransfected with cGAS plus STING and either EV or the indicated UL138 expression construct. Fold induction of IFN-β promoter luciferase activity relative to the control with no cGAS or STING is shown (*n* = 4). (I) Representative Western blot for the indicated proteins from lysates from panel H. Tubulin served as a loading control (*n* = 4). (J) Quantitation of STING levels from panel I normalized to tubulin levels and shown relative to the EV control from the same blot (*n* = 4). (K) IFN-β promoter luciferase reporter assays as in panel A from 293T cells cotransfected with the indicated STING expression construct and either EV or the UL138-HA expression construct. Fold induction of IFN-β promoter luciferase activity relative to the no-STING control is shown (*n* = 4). (L) Representative Western blot for the indicated proteins from lysates from panel K. GAPDH served as a loading control (*n* = 4). (M) Quantitation of STING levels from panel L normalized to GAPDH levels and shown relative to the EV control from the same blot (*n* = 4). (N) 293T cells cotransfected with cGAS plus STING expression vectors and either EV or increasing amounts of UL138 expression vector for 48 h. A representative Western blot is shown for the indicated proteins. GAPDH served as a loading control (*n* = 3). (O) Quantitation of STING protein levels from panel N normalized to GAPDH levels and shown relative to the no-UL138 control from the same blot (*n* = 3). (P) 293T cells cotransfected with cGAS plus STING expression vectors and either EV or the UL138 expression vector for 48 h and treated with either DMSO vehicle control, one of the proteasome inhibitors lactacystin (Lacta) or MG132, or the lysosomal inhibitor concanamycin A (CcmA). A representative Western blot is shown for the indicated proteins. Tubulin served as a loading control (*n* = 3). (Q) Quantitation of STING levels from panel P normalized to tubulin levels and shown relative to the no-UL138 DMSO control from the same blot (*n* = 3). (R) 293T cells cotransfected with STING and either EV or the UL138-HA expression construct for 48 h. A representative Western blot is shown for indicated proteins. GAPDH served as a loading control (*n* = 4). (S) Quantitation of protein levels for the indicated protein from panel R normalized to GAPDH levels and shown relative to the EV control from the same blot (*n* = 4). (T) 293T cells cotransfected with cGAS plus STING and either EV or the indicated UL138 expression construct for 48 h and analyzed for STING transcripts by RT-qPCR. STING transcripts were normalized to GAPDH and are shown relative to EV from the same experiment (*n* = 3). Bar graphs show the means ± SEM from the indicated number of biological replicates.

After observing the colocalization and interaction of UL138 and STING, we next asked if UL138 affected STING function. STING function is commonly quantitated by an interferon beta (IFN-β) reporter assay (30–33). As expected, we found that transfection of either STING or cGAS alone was not sufficient to robustly activate an IFN-β promoter reporter construct, but cotransfection with both cGAS and STING led to an ∼6-fold induction of IFN-β promoter activity (Fig. 2A). UL138 suppressed the cGAS/STING-mediated induction of IFN-β promoter activity, as did the known HCMV-encoded STING antagonist pp71 (UL82) (19) (Fig. 2A). UL138 inhibited cGAS/STING-mediated activation of the IFN-β promoter when C-terminally tagged with either a hemagglutinin (HA) or tandem FLAG epitope tag or when untagged (wild type [WT]) (Fig. 2E). In contrast, two loss-of-function alleles of UL138 cloned from different published recombinant TB40/E strain viruses, either a deletion of amino acids 40 to 154 (Δ40–154) (34) or conversion of the methionine 16 codon to a stop codon (M16stop) (35, 36), failed to suppress cGAS/STING-mediated activation of the IFN-β promoter (Fig. 2E). UL138 encodes four Golgi sorting motifs that cooperate to maintain its Golgi localization (37). A UL138 mutant in which the second tyrosine sorting motif is inactivated (mY2) failed to suppress cGAS/STING-mediated activation of the IFN-β promoter in 293T cell transfections (Fig. 2H). We conclude that HCMV UL138 suppresses cGAS/STING-mediated activation of the IFN-β promoter.

STING can be activated by cGAS-synthesized 2′3′-cGAMP or by mutation. For example, the naturally occurring SAVI (V155M) allele (38) and the artificially created R284M allele (39) are constitutively active STING mutants. UL138 was able to inhibit the ability of STING-V155M and STING-R284M to activate the IFN-β promoter reporter in the absence of cGAS in transfected 293T cells (Fig. 2K). An inactive STING mutant (S366A) failed to activate the IFN-β promoter reporter. We conclude that UL138 inhibits the cGAS/STING pathway downstream of activated STING.

### HCMV UL138 induces lysosomal degradation of STING.

Western blot examination (Fig. 2B) of cellular lysates from reporter assays (Fig. 2A) revealed that STING accumulated to lower steady-state levels in the presence of UL138 (Fig. 2C), while cGAS levels were not significantly different in the absence or presence of UL138 (Fig. 2D). In contrast, pp71 did not substantially affect the steady-state levels of either cGAS or STING. Loss-of-function UL138 alleles from published TB40/E strain recombinant viruses did not significantly decrease STING steady-state levels (Fig. 2F and G). UL138-mY2, unable to suppress cGAS/STING-mediated activation of the IFN-β promoter, also did not significantly decrease STING steady-state levels (Fig. 2I and J). However, the steady-state levels of the constitutively active forms of STING (V155M and R284M) were reduced by UL138 (Fig. 2L and M).

Transfection of increasing amounts of UL138 led to corresponding decreases in the steady-state levels of cotransfected STING (Fig. 2N and O), suggesting that UL138 affects STING protein levels in a dose-dependent manner. STING protein levels could be stabilized in the presence of UL138 by addition of the lysosome inhibitor concanamycin A but not by addition of proteasome inhibitors lactacystin or MG132 (Fig. 2P and Q), indicating that UL138-mediated destabilization of STING occurs via the lysosome, similar to what has been observed for another UL138 target, MRP1 (40). UL138 did not induce the degradation of the Golgi resident GM130 protein, the ER resident calnexin protein, or the nuclear resident histone H3 protein (Fig. 2R and S). Finally, in 293T cell transfections, cotransfected UL138 had a minimal effect on cotransfected STING transcript levels that was not statistically significant (Fig. 2T). We conclude that UL138 reduces the steady-state levels of the STING protein by promoting its lysosomal degradation.

### HCMV UL138 colocalizes with and interacts with TBK1 and inhibits IRF3 phosphorylation, but it does not substantially reduce TBK1 steady-state levels.

**FIG 3:**
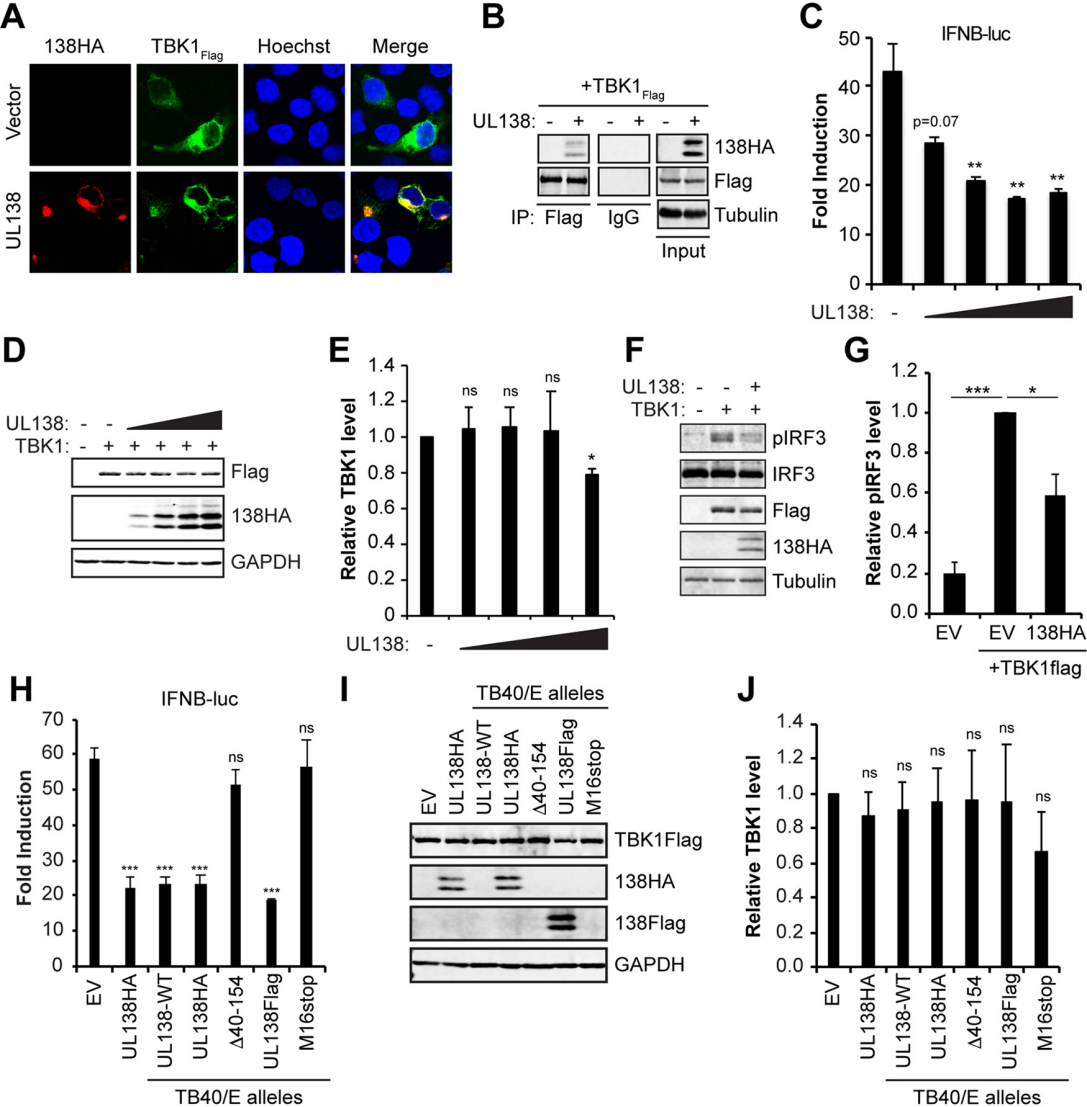
HCMV UL138 colocalizes with, interacts with, and inhibits TBK1 but does not reduce its steady-state levels. (A) 293T cells cotransfected with an expression construct for FLAG-tagged TBK1 and either empty vector or HA-tagged UL138 for 48 h and stained for HA-tagged UL138 and FLAG-tagged TBK1. Nuclei were counterstained with Hoechst (*n* = 3). (B) Coimmunoprecipitation experiments with 293T cells cotransfected with expression constructs for FLAG-tagged TBK1 and either empty vector (-) or HA-tagged UL138 (+) for 48 h, with Western blotting performed for the indicated proteins (*n* = 3). (C) IFN-β promoter luciferase assays in 293T cells cotransfected with expression constructs for TBK1 and either EV (-) or increasing amounts of UL138 for 48 h. Fold induction of IFN-β promoter luciferase activity relative to the no-TBK1 control is shown (*n* = 3). (D) Representative Western blot for the indicated proteins from lysates from panel C. GAPDH served as a loading control (*n* = 3). (E) Quantitation of TBK1 levels from panel D normalized to GAPDH levels and shown relative to the TBK1-only transfected control from the same blot (*n* = 3). (F) 293T cells were cotransfected with the indicated expression constructs for 48 h, with Western blotting performed for the indicated proteins (*n* = 4). Tubulin served as a loading control. (G) Quantitation of phosphorylated IRF3 levels from panel F normalized to total IRF3 levels and shown relative to the TBK1-only transfected control from the same blot (*n* = 4). (H) IFN-β promoter luciferase reporter assays as in panel C from 293T cells transfected with expression constructs for TBK1 and either EV (-) or the indicated UL138 expression construct made from recombinant TB40/E viruses. Fold induction of IFN-β promoter luciferase activity relative to the no-TBK1 control is shown (*n* = 4). (I) Representative Western blot for the indicated proteins from lysates from panel H. GAPDH served as a loading control (*n* = 4). (J) Quantitation of TBK1 protein levels from panel I normalized to GAPDH levels and shown relative to the TBK1-only transfected control from the same blot (*n* = 4). Bar graphs show the means ± SEM from the indicated number of biological replicates.

At the Golgi, STING interacts with and activates TBK1, leading to IRF3 phosphorylation and IFN-β promoter activation (41, 42). Given the interaction between UL138 and STING at the Golgi, and the negative effect of UL138 on STING-mediated activation of the IFN-β promoter reporter, we next asked if UL138 colocalized with, interacted with, or inhibited TBK1. We found that UL138 colocalized with (Fig. 3A) and coimmunoprecipitated (Fig. 3B) TBK1. Transfection of 293T cells with TBK1 activated an IFN-β promoter reporter, and this activation was impaired upon cotransfection with increasing amounts of UL138 (Fig. 3C and D), indicating that UL138 inhibits TBK1-mediated activation of the IFN-β promoter.

STING induces IRF3 activation through TBK1-mediated phosphorylation (43). How STING activates NF-κB is less clear. Having shown that UL138 inhibits activated STING- and TBK1- mediated activation of the IFN-β promoter, we next asked if UL138 inhibited TBK1-mediated IRF3 phosphorylation. In 293T cells transfected with TBK1, cotransfected UL138 reduced the accumulation of phosphorylated IRF3 (Fig. 3F and G).

In contrast to its effects on STING, UL138 did not consistently or considerably alter the steady-state levels of TBK1 despite a modest reduction under certain assay conditions (Fig. 3D and E). Both tagged and untagged alleles of full-length UL138 inhibited the ability of TBK1 to activate the IFN-β promoter, but loss-of-function alleles failed to suppress TBK1-mediated activation of the IFN-β promoter, and none of them reduced TBK1 steady-state levels (Fig. 3H to J). We conclude that UL138 interacts with both STING and TBK1 and inhibits the ability of the cGAS/STING/TBK1 pathway to induce IRF3 phosphorylation and activate the IFN-β promoter.

### HCMV UL138 acts upstream of NF-κB and IRF3.

**FIG 4:**
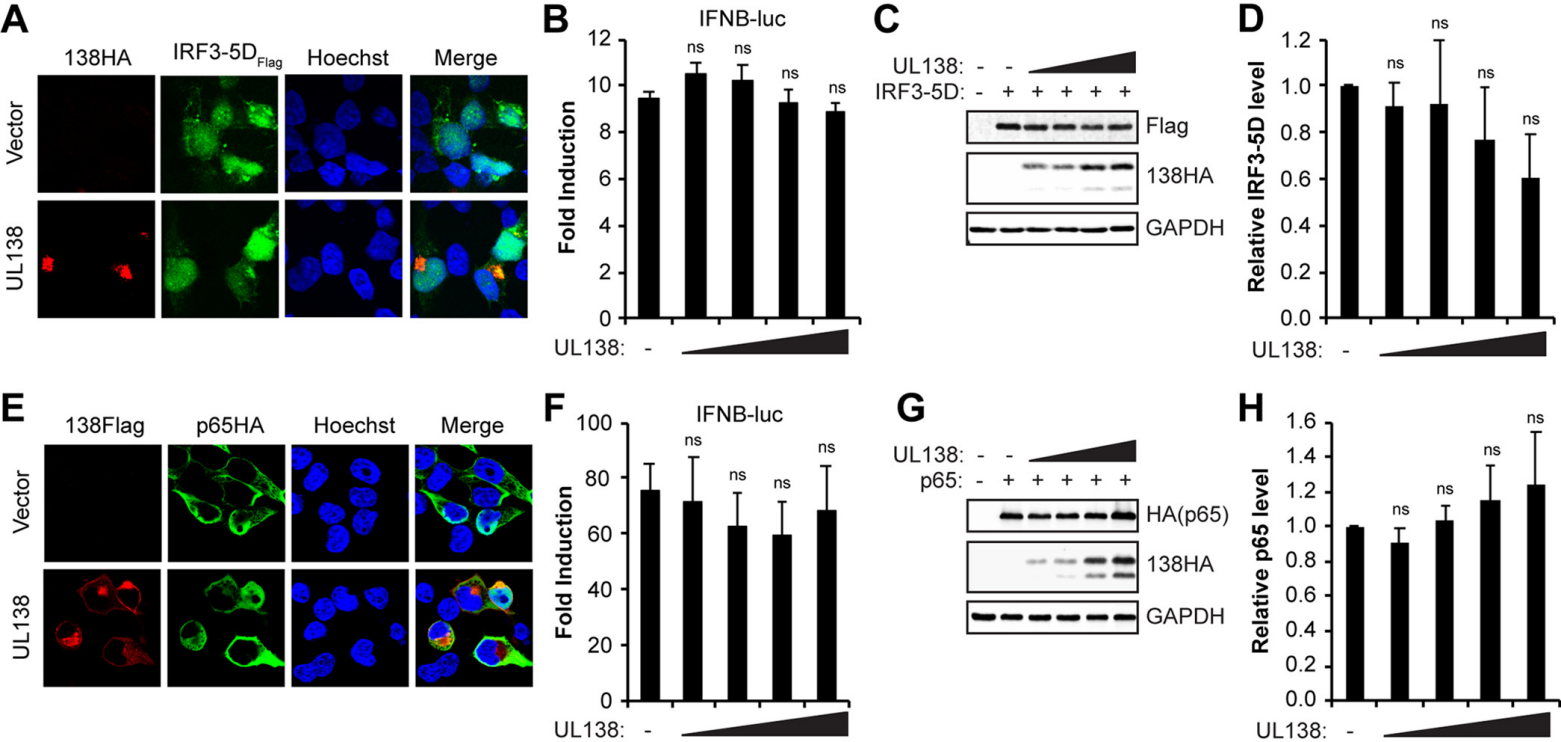
HCMV UL138 acts upstream of NF-κB and IRF3. (A) 293T cells cotransfected with expression constructs for IRF3-5D and either empty vector or UL138 for 48 h and stained for HA-tagged UL138 and FLAG-tagged IRF3-5D. Nuclei were counterstained with Hoechst (*n* = 3). (B) IFN-β promoter luciferase assays in 293T cells cotransfected with IRF3-5D and either empty vector (-) or increasing amounts of the UL138-HA expression construct for 48 h. Fold induction of IFN-β promoter luciferase activity relative to the no-IRF3-5D control is shown (*n* = 3). (C) Western blot for the indicated proteins from lysates from panel B (*n* = 3). (D) Quantitation of IRF3-5D levels from panel C normalized to GAPDH levels and shown relative to IRF3-5D-only transfected controls from the same blot (*n* = 3). (E) 293T cells cotransfected with NF-κB p65/RelA and either empty vector or FLAG-tagged UL138 for 48 h and stained for HA-tagged p65/RelA and FLAG-tagged UL138. Nuclei were counterstained with Hoechst (*n* = 3). (F) IFN-β promoter luciferase assays in 293T cells cotransfected with p65/RelA and either empty vector (-) or increasing amounts of the UL138-HA expression construct for 48 h. Fold induction of IFN-β promoter luciferase activity relative to the no-p65/RelA control is shown (*n* = 3). (G) Western blot for the indicated proteins from lysates from panel F (*n* = 3). (H) Quantitation of p65/RelA levels from panel G normalized to GAPDH levels and shown relative to the p65/RelA-only transfected controls from the same blot (*n* = 3). Bar graphs show the means ± SEM from the indicated number of biological replicates.

Activation of the cGAS/STING/TBK1 pathway ultimately leads to phosphorylation (and activation) of IRF3 and NF-κB (43, 44), each of which have roles during the induction of IFN-β (45, 46). Thus, we next asked if UL138 could interfere with the activity of IRF3 and/or NF-κB. In cotransfected 293T cells, UL138 did not strongly colocalize with or prevent the nuclear accumulation of IRF3-5D, a constitutively active IRF3 derivative (Fig. 4A). Furthermore, UL138 had no effect on the ability of overexpressed IRF3-5D to activate the IFN-β promoter (Fig. 4B), nor did it affect the steady-state levels of IRF3-5D (Fig. 4C and D). Similarly, UL138 did not strongly colocalize with the p65RelA subunit of NF-κB (Fig. 4E), nor did it affect the ability of overexpressed p65RelA to activate the IFN-β promoter (Fig. 4F) or alter p65RelA steady-state protein levels (Fig. 4G and H). Thus, UL138 is unable to inhibit the cGAS/STING/TBK1 pathways downstream of IRF3 or NF-κB activation. Taken together, these results lead us to conclude that UL138 inhibits the cGAS/STING/TBK1 pathway downstream of activated STING but upstream of IRF3 and NF-κB activation.

### HCMV UL138 inhibits IFN-β promoter activity in cell types relevant to HCMV lytic and latent infection.

**FIG 5:**
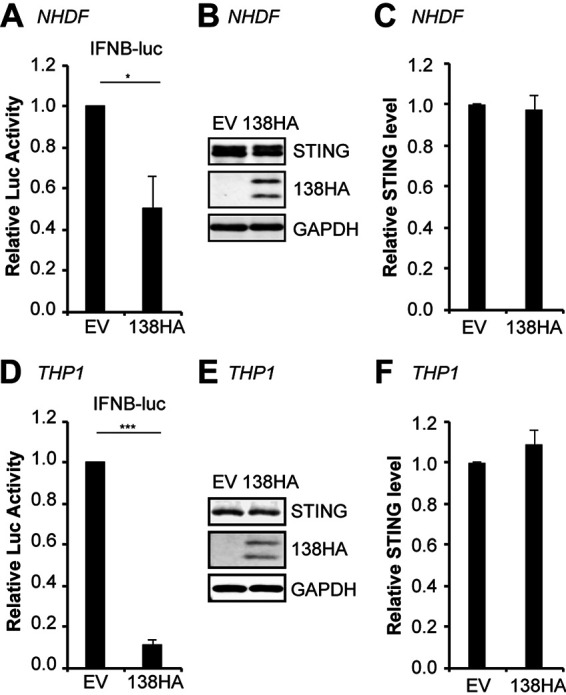
HCMV UL138 inhibits IFN-β promoter activity in cell types relevant to HCMV lytic and latent infection. IFN-β promoter reporter assays in NHDF (A to C) or THP-1 (D to F) cells cotransfected with IFN-β promoter-driven firefly luciferase and either EV or UL138. (A and D) Fold induction of IFN-β promoter luciferase activity relative to EV control is shown (*n* = 4). (B and E) Representative Western blot for the indicated proteins from the luciferase assays. GAPDH served as a loading control (*n* = 4). (C and F) Quantitation of STING levels from panels B and E normalized to GAPDH levels and shown relative to the EV control from the same blot (*n* = 4). Bar graphs show the means ± SEM from the indicated number of biological replicates.

We next asked whether UL138 could inhibit the IFN-β promoter in cell types in which HCMV initiates a productive, lytic infection (normal human dermal fibroblasts [NHDFs]) or establishes latency (THP-1 monocytes). In both transfected fibroblasts (Fig. 5A and B) and THP-1 monocytes (Fig. 5D and E), UL138 suppressed the IFN-β promoter reporter. We did not observe statistically significant decreases in the steady-state levels of endogenous STING during reporter assays in fibroblasts (Fig. 5B and C) or THP-1 cells (Fig. 5E and F), likely due to the relatively low transfection efficiency of these cells. We conclude that UL138 inhibits the cGAS/STING/TBK1 innate immune response in 293T cells, in fibroblasts (in which HCMV infection is lytic), and in THP-1 cells (in which HCMV infection is latent).

### HCMV UL138 colocalizes and interacts with STING during HCMV productive infection with a laboratory strain virus.

**FIG 6:**
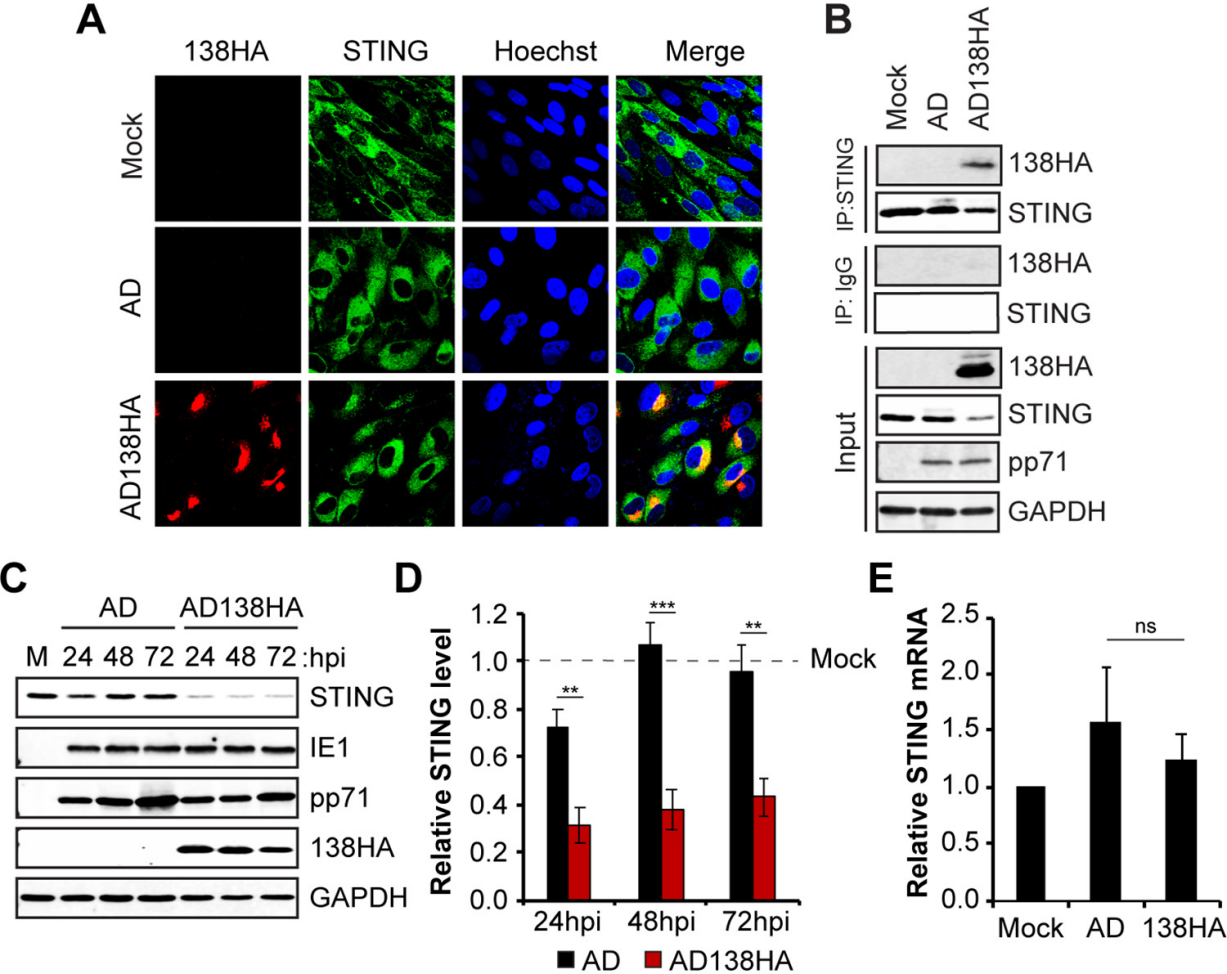
HCMV UL138 colocalizes and interacts with STING during HCMV productive infection with a laboratory strain virus. (A) NHDFs mock infected or infected with wild-type AD169 (AD) or AD169-UL138-HA (AD138HA) virus at an MOI of 1 for 48 h were stained for HA-tagged UL138 and endogenous STING. Nuclei were counterstained with Hoechst (*n* = 3). (B) Coimmunoprecipitation experiments with NHDFs mock infected or infected with the indicated virus at an MOI of 3 for 24 h, with Western blotting performed for the indicated proteins (*n* = 3). (C) NHDFs mock infected (M) or infected with the indicated virus at an MOI of 1 and harvested at the indicated hour postinfection (hpi), with Western blotting performed for the indicated proteins. GAPDH served as a loading control (*n* = 5). (D) Quantitation of STING protein levels from panel C normalized to GAPDH levels and shown relative to the mock-infected control from the same blot (*n* = 5). (E) NHDFs mock infected or infected with the indicated virus at an MOI of 1 for 24 h were analyzed for STING transcripts by RT-qPCR. STING transcript levels were normalized to GAPDH transcripts and are shown relative to mock-infected cells from the same experiment (*n* = 3). Bar graphs show the means ± SEM from the indicated number of biological replicates.

We used a return-of-function approach as a first examination of the effects of UL138 on the cGAS/STING/TBK1 pathway and IFN-β mRNA accumulation during HCMV infection. During extended passage, the laboratory-adapted strain of HCMV AD169 (47) suffered an ∼15-kb deletion of the genomic region designated ULb′, which contains ∼19 genes, including the locus that encodes UL138 (48–51). Thus, AD169 represents a natural, loss-of-function, UL138-null virus. UL138 function has previously been restored to AD169 in a recombinant revertant (AD169-UL138-HA) in which an HA-tagged allele of UL138 was added back to AD169 near its ancestral locus and under the control of its native putative promoter (34). We used this virus to infect fibroblasts using parental AD169 as a control and found that UL138 synthesized by AD169-UL138-HA colocalized with (Fig. 6A) and coimmunoprecipitated (Fig. 6B) endogenous STING. Fibroblasts infected with AD169-UL138-HA also showed significantly reduced steady-state levels of STING protein compared to those in mock-infected cells or cells infected with the parental AD169 (Fig. 6C and D), indicating that UL138 is sufficient to destabilize STING in the context of AD169 infection of fibroblasts. STING transcript levels were not affected by AD169-UL138-HA infection (Fig. 6E). We conclude that UL138 colocalizes with, interacts with, and destabilizes STING during a productive infection initiated by a laboratory strain virus.

### A UL138-positive laboratory strain virus shows reduced IFN-β transcript accumulation compared to that of wild-type virus during infection of either fibroblasts or myeloid cells.

**FIG 7:**
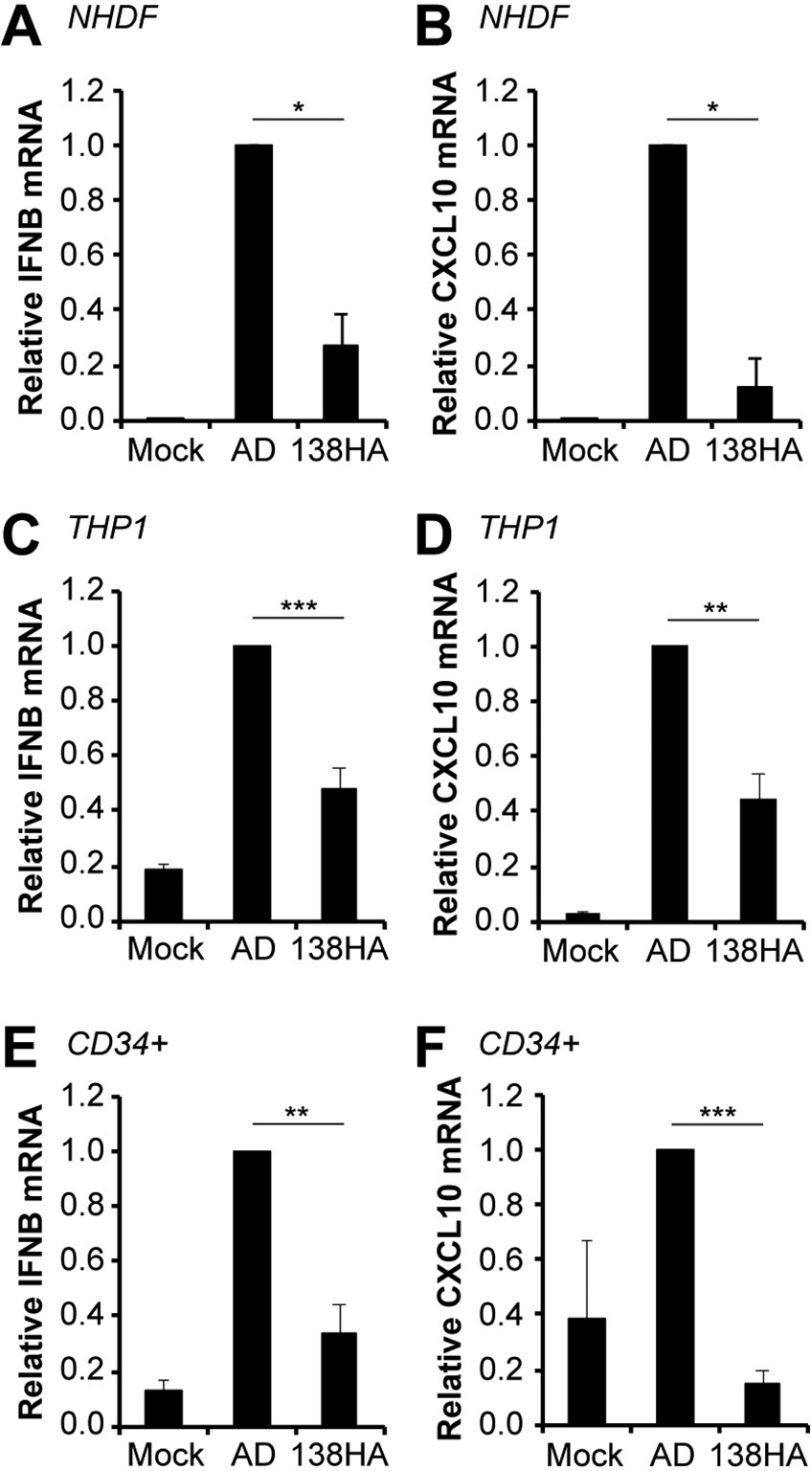
A UL138-positive laboratory strain virus shows reduced IFN-β and CXCL10 transcript accumulation compared to wild-type virus during infection of either fibroblasts or myeloid cells. (A and B) NHDFs mock infected or infected with wild-type AD169 or AD169-UL138-HA virus at an MOI of 1 for 24 h were analyzed for IFN-β (A) and CXCL10 (B) transcripts by RT-qPCR. Transcript levels relative to AD169-infected cells are shown (*n* = 3). (C and D) THP-1 monocytes mock infected or infected with the indicated virus at an MOI of 1 for 24 h were analyzed for IFN-β (C) and CXLC10 (D) transcripts by RT-qPCR. Transcript levels relative to AD169-infected cells are shown (*n* = 6). (E and F) Primary CD34^+^ HPCs were mock infected or infected with the indicated virus at an MOI of 1 for 24 h and were analyzed for IFN-β (E) and CXCL10 (F) transcripts by RT-qPCR. Transcript levels relative to AD169-infected cells are shown (*n* = 5). Bar graphs show the means ± SEM from the indicated number of biological replicates.

Inactivation of the STING pathway should decrease the steady-state accumulation of IFN-β mRNA. Thus, restoring UL138 to AD169 should reduce IFN-β mRNA accumulation induced by this recombinant viral strain. Indeed, in productively infected fibroblasts, we quantitated significantly fewer IFN-β (Fig. 7A) and CXCL10 (Fig. 7B) transcripts in cells infected with AD169 expressing UL138 compared to wild-type AD169. We conclude that UL138 inhibits innate immune signaling during HCMV productive infection of fibroblasts with a laboratory strain virus despite the presence of nine additional virally encoded cGAS/STING/TBK1 pathway antagonists.

A feature unique to UL138 and distinct from the nine previously identified cGAS/STING/TBK1 pathway antagonists encoded by HCMV is that UL138 is expressed and acts during latency in myeloid cells. To determine if UL138 neutralized the innate immune response in myeloid cells, we infected THP-1 monocytes with either AD169 or AD169 restored to encode UL138. We quantitated significantly fewer IFN-β (Fig. 7C) and CXCL10 (Fig. 7D) transcripts in THP-1 cells infected with AD169 expressing UL138 than with wild-type AD169. In addition to monocytes, HCMV latently infects CD34^+^ hematopoietic progenitor cells (24, 52, 53). *In vitro*, primary CD34^+^ cells are considered to provide greater physiological relevance than transformed THP-1 cells. When we analyzed IFN-β transcripts in CD34^+^ cells, we found results identical to those for fibroblasts and THP-1 cells. Primary CD34^+^ cells infected with AD169 expressing UL138 accumulated significantly fewer IFN-β (Fig. 7E) and CXCL10 (Fig. 7F) transcripts than did cells infected with wild-type AD169. We conclude that UL138 reexpressed from a laboratory strain virus suppresses innate immune signaling and IFN-β mRNA accumulation in myeloid cells.

### HCMV UL138 colocalizes and interacts with STING during HCMV productive infection with a clinical strain virus.

**FIG 8:**
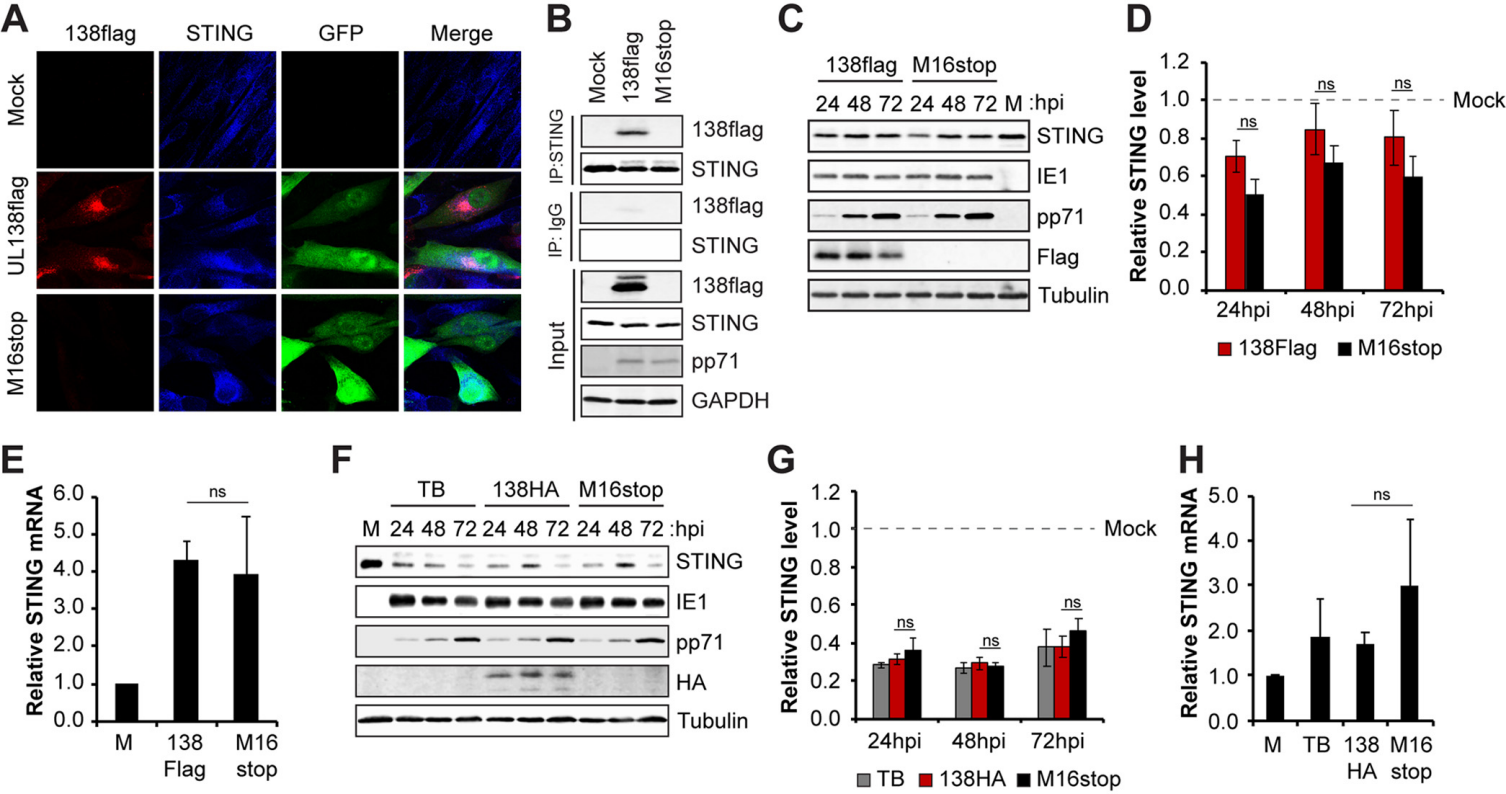
HCMV UL138 colocalizes and interacts with STING during HCMV productive infection with a clinical strain virus. (A) NHDFs mock infected or infected with the indicated TB40/E-GFP virus at an MOI of 1 for 48 h were stained for FLAG-tagged UL138 and endogenous STING. Nuclei were counterstained with Hoechst. GFP served as a marker for viral infection (*n* = 3). (B) Coimmunoprecipitation experiments with NHDFs mock infected or infected with the indicated virus at an MOI of 3 for 24 h, with Western blotting performed for the indicated proteins (*n* = 3). (C) NHDFs mock infected for 24 h or infected with the indicated virus at an MOI of 1 and harvested at the indicated hour postinfection, with Western blotting performed for the indicated proteins. Tubulin served as a loading control (*n* = 3). (D) Quantitation of STING protein levels from panel C normalized to tubulin levels and shown relative to mock-infected controls from the same blot (*n* = 3). (E) NHDFs mock infected or infected with the indicated virus at an MOI of 1 for 24 h were analyzed for STING transcripts by RT-qPCR. STING transcript levels were normalized to GAPDH transcripts and are shown relative to mock-infected cells from the same experiment (*n* = 3). (F) NHDFs mock infected for 24 h or infected with the indicated virus at an MOI of 1 and harvested at the indicated hour postinfection, with Western blotting performed for the indicated proteins. Tubulin served as a loading control (*n* = 3). (G) Quantitation of STING protein levels from lysates used for panel F normalized to tubulin levels and shown relative to mock-infected controls from the same blot (*n* = 3). (H) NHDFs mock infected or infected with the indicated virus at an MOI of 1 for 24 h were analyzed for STING transcripts by RT-qPCR. STING transcript levels were normalized to GAPDH transcripts and are shown relative to mock-infected cells from the same experiment (*n* = 3). Bar graphs show the means ± SEM from the indicated number of biological replicates.

Low-passage clinical strains of HCMV encode UL138 and are considered by some to be more physiologically relevant than laboratory strains. Therefore, we asked whether UL138 could colocalize and interact with STING during productive infection with the clinical strain virus TB40/E. We found that UL138 synthesized by TB40/E colocalized with (Fig. 8A) and coimmunoprecipitated (Fig. 8B) endogenous STING upon infection of fibroblasts. We conclude that UL138 colocalizes and interacts with STING during a productive infection initiated by a clinical strain virus.

While STING levels were reduced in TB40/E-infected fibroblasts compared to a mock infection (Fig. 8D), there was little difference in STING levels between fibroblasts infected with a virus encoding a UL138 allele with an added carboxy-terminal 3× FLAG epitope (TB40/E-green fluorescent protein [GFP]-UL138-FLAG) and one encoding an untagged UL138 allele with an inserted stop codon at position 16 (TB40/E-GFP-UL138-M16stop) (Fig. 8C and D). STING transcript levels were not affected by infection with these TB40/E-based viruses (Fig. 8E). We were surprised to see little difference in STING levels between the TB40/E-based UL138-FLAG and M16stop viruses, because we saw significant UL138-mediated STING degradation during AD169 fibroblast infection (Fig. 6C and D). To independently retest STING degradation during TB40/E infection, we created two new TB40/E recombinant viruses based on the same parental strain (TB40/E-GFP-WT in which UL138 is untagged) that are either tagged at the carboxy terminus with the HA epitope (TB40/E-GFP-UL138-HA) or tagged at the carboxy terminus with the HA epitope but with an inserted STOP codon at position 16 (TB40/E-GFP-UL138-HA-M16stop). With these genetically matched viruses in which the inability of the stop codon mutant to produce UL138 can be detected by Western blotting with an HA antibody, we found STING levels reduced during TB40/E infection of fibroblasts, but the reduction was not dependent upon the presence of UL138 (Fig. 8F and G). STING transcript levels were not affected by infection with these TB40/E-based viruses (Fig. 8H). Because TB40/E-based UL138 alleles can degrade STING during transfections (Fig. 2F and G), it seems likely that TB40/E may encode additional STING regulators not expressed during AD169 infections (see Discussion).

### UL138-null clinical strain viruses show enhanced IFN-β transcript accumulation during both lytic and latent infections compared to that of viruses expressing functional UL138.

**FIG 9:**
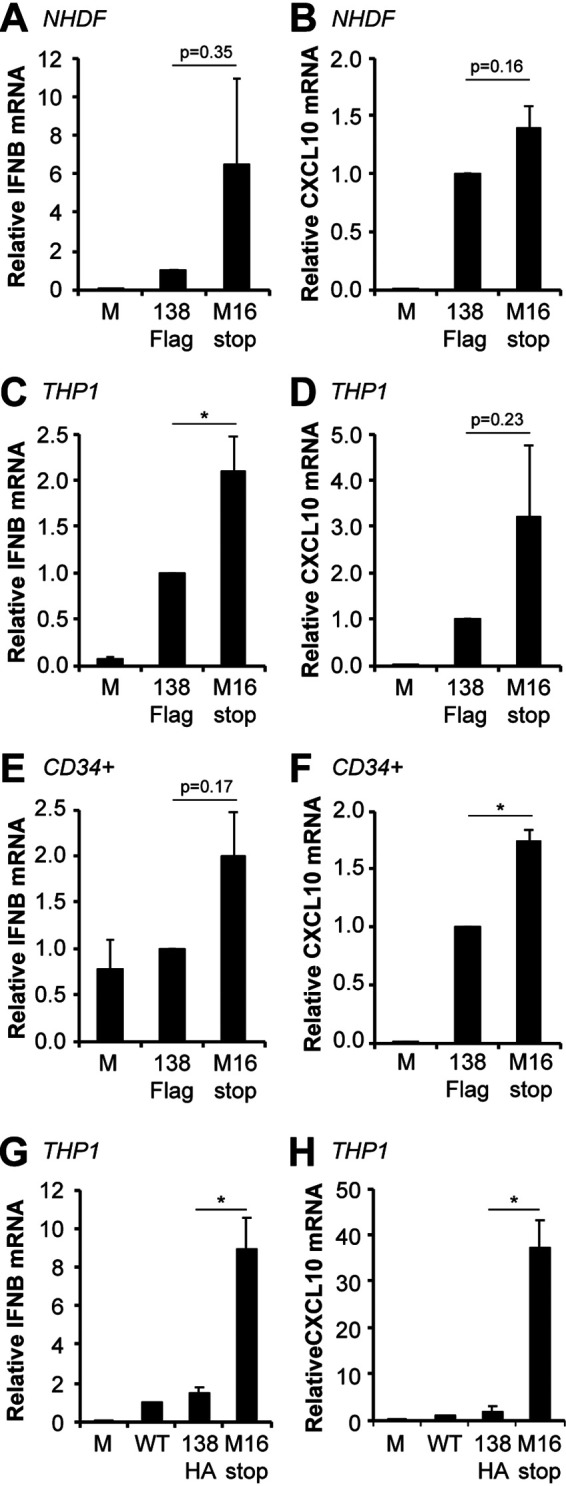
A UL138-null clinical strain virus shows enhanced IFN-β and CXCL10 transcript accumulation compared to wild-type virus during both lytic and latent infections. (A and B) NHDFs mock infected or infected with TB40/E-GFP-UL138-FLAG (138Flag) or TB40/E-GFP-UL138-M16stop (M16stop) virus at an MOI of 1 for 24 h were analyzed for IFN-β (A) and CXCL10 (B) transcripts by RT-qPCR. Transcript levels relative to 138Flag-infected cells are shown (*n* = 3). (C and D) THP-1 monocytes mock infected or infected with the indicated virus at an MOI of 1 for 24 h were analyzed for IFN-β (C) and CXLC10 (D) transcripts by RT-qPCR. Transcript levels relative to 138Flag-infected cells are shown (*n* = 5). (E and F) Primary CD34^+^ HPCs mock infected or infected with the indicated virus at an MOI of 1 for 24 h were analyzed for IFN-β (E) and CXCL10 (F) transcripts by RT-qPCR. Transcript levels relative to 138Flag-infected cells are shown (*n* = 3). (G and H) THP-1 monocytes mock infected or infected with parental TB40/E-GFP (WT), TB40/E-GFP-UL138-HA (138HA), or TB40/E-GFP-UL138-HA-M16stop (M16stop) virus at an MOI of 1 for 24 h were analyzed for IFN-β (G) and CXCL10 (H) transcripts by RT-qPCR. Transcript levels relative to WT-infected cells are shown (*n* = 3). Bar graphs show the means ± SEM from the indicated number of biological replicates.

Inactivation of the STING pathway should decrease the steady-state accumulation of the IFN-β mRNA. Thus, removing UL138 from TB40/E should increase IFN-β mRNA accumulation induced by this viral strain. Indeed, in productively infected fibroblasts, we quantitated more IFN-β (Fig. 9A) and CXCL10 (Fig. 9B) transcripts in cells infected with UL138-null TB40/E than with TB40/E in which the UL138 allele is wild type except for the carboxy-terminal FLAG tag. We conclude that UL138 inhibits innate immune signaling during HCMV productive infection of fibroblasts with a clinical strain virus despite the presence of at least nine additional virally encoded cGAS/STING/TBK1 pathway antagonists.

**FIG 10:**
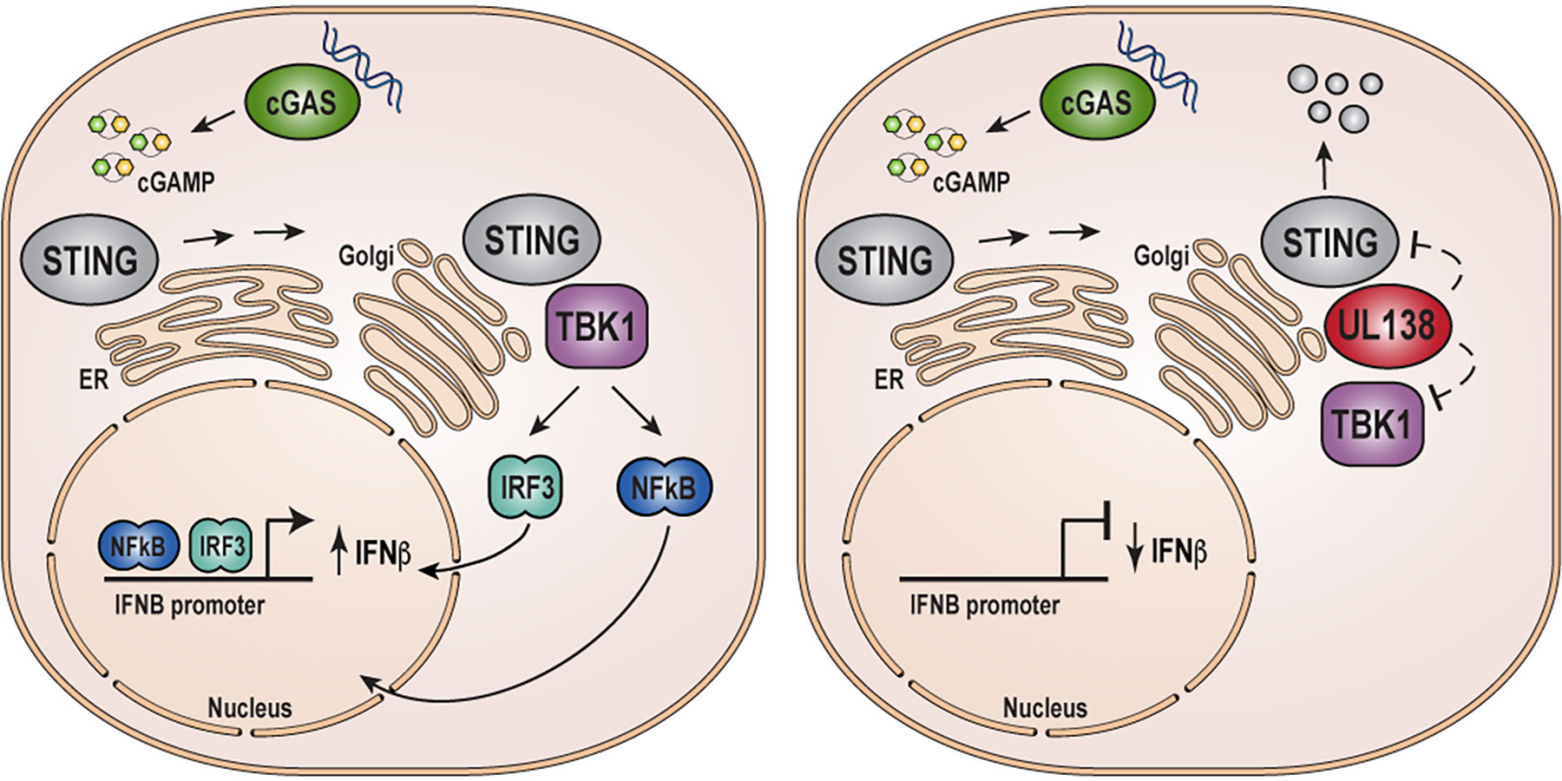
HCMV UL138 protein inhibits the STING pathway and reduces IFN-β mRNA accumulation during lytic and latent infections. Shown is a model for the activation of IFN-β transcription by the cGAS/STING/TBK1 pathway in the absence or presence of HCMV UL138.

The use of a clinical strain allows for the examination of a true latent infection in myeloid cells. We quantitated significantly more IFN-β (Fig. 9C) and more CXCL10 (Fig. 9D) transcripts in THP-1 cells latently infected with UL138-null TB40/E than with TB40/E in which the UL138 allele is wild type except for the carboxy-terminal FLAG tag. Similarly, primary CD34^+^ cells infected with UL138-null TB40/E accumulated higher levels of IFN-β (Fig. 9E) and significantly higher levels of CXCL10 (Fig. 9F) transcripts than did those infected with TB40/E in which the UL138 allele is wild type except for the carboxy-terminal FLAG tag. Finally, we used our newly created, genetically matched TB40/E viruses to show that significantly more IFN-β (Fig. 9G) and CXCL10 (Fig. 9H) transcripts accumulate in THP-1 cells in the absence of UL138 than in its presence. We conclude that UL138 expressed from a clinical strain virus suppresses innate immune signaling and IFN-β mRNA accumulation during latency. In total, we conclude that HCMV UL138 inhibits the cGAS/STING/TBK1 pathway during viral productive infections and latency (Fig. 10).

## DISCUSSION

The induction of IFN-β is important for controlling viral infections. In particular, the cGAS/STING/TBK1 foreign DNA sensing pathway is a major contributor to the IFN response during infection with DNA viruses (54). HCMV, a double-stranded DNA virus, has the largest genome of any known human virus and therefore presents high levels of this pathway’s ligand during infection. Thus, it is remarkable, but perhaps not surprising, that HCMV possesses at least 10 genes that produce protein inhibitors of the cGAS/STING/TBK1 pathway. In addition to genes for nine previously described proteins (UL31, UL35, UL42, UL48, UL82 [pp71], UL83 [pp65], UL94, UL122 [IE2], and Us9) and the newly defined cGAS/STING/TBK1 inhibitor identified here (UL138), low-passage-number strains of HCMV possess an additional ∼18 genes (48–51) that have yet to be screened for pathway inhibition. The smaller decreases in pathway output with low-passage-number TB40/E compared to high-passage-number AD169 restored for UL138 expression seen in this study may indicate that additional TB40/E genes beyond UL138 may target this pathway. Thus, the number of HCMV-encoded inhibitors of the cGAS/STING/TBK1 pathway seems likely to climb even higher.

With the myriad cell types HCMV infects (8, 9), as well as the three different infection programs (productive, persistent, and latent) (26), encoding multiple cGAS/STING/TBK1 pathway inhibitors likely provides flexibility and insurance. Even RNA viruses, with much smaller genomes, encode multiple cGAS/STING/TBK1 pathway inhibitors (54), testifying to the importance of this pathway during viral infection. Furthermore, HCMV encodes multiple inhibitors of other independent innate immune pathways (e.g., RIG-I) that suppress their ability to induce IFN production, further contributing to redundancy. How the combination of innate immune inhibitors, specific infection programs, and cell type differences cooperate to modulate innate immunity and viral infection will be challenging but important to discover and may not be completely mimicked by reductionist studies.

The exact mechanism through which UL138 inhibits the cGAS/STING/TBK1 pathway is unclear, but the restriction occurs after STING activation but prior to the function of downstream transcription factors IRF3 and NF-κB. Because UL138 also interacts with and inhibits TBK1, a direct effect on TBK1 function or binding to STING seems possible. Like MRP1, UL138 induces the degradation of STING by a lysosomal pathway (40). While an acidic cluster dileucine sorting motif in UL138 is required for MRP1 degradation (37), one of the tyrosine sorting motifs (mY2) appears to direct STING to the lysosome for degradation during transfection. Why different sorting motifs mediate these independent degradation events remains to be investigated. While the ability of UL138 to promote the degradation of STING almost certainly contributes to pathway inhibition, it does not appear to be the single, essential functional outcome.

STING levels are reduced after activation by a dedicated regulatory mechanism that downregulates pathway function to moderate the immune response (55). Furthermore, both the lytic-phase-only IE2 protein (24) and UL138, which is expressed during both lytic infection and latency (27, 28), reduce STING steady-state levels (14) (Fig. 2C, G, J, M, O, Q, and S and Fig. 6D). However, UL138 remains dispensable for STING downregulation during TB40/E infections, perhaps indicating that additional TB40/E-encoded proteins can also downregulate STING and suppress IFN-β accumulation. How each of these multiple STING-decreasing mechanisms contributes to STING levels and immune responses in HCMV-infected cells remains to be determined.

The redundancy provided by the multiple cGAS/STING/TBK1 and other IFN-producing pathway inhibitors encoded by HCMV means that inactivating a single STING antagonist amid the background of additional pathway inhibitors results in modest quantitative effects on pathway output. Indeed, of the nine known HCMV STING antagonists, seven have had their effects on IFN-β pathway suppression during infection quantitated by either small interfering RNA (siRNA)-mediated knockdown or with viral mutants, and all seven have shown only small increases in IFN-β mRNA induction in the absence of a single STING antagonist (11–13, 18, 19, 21, 22), similar in magnitude to effects observed in this study. Thus, our quantitative findings on the effects of UL138 on this pathway align well with previous studies and with the concept of functional redundancy.

Not surprisingly, we observed the highest magnitude of effects of UL138 on this pathway during latent infection of incompletely differentiated myeloid cells in which the nine previously identified pathway inhibitors are not expressed. Although significant work has gone into understanding innate immune signaling and mechanisms of viral antagonism during productive HCMV replication (56–59), considerably less is known about viral countermeasures to innate immune sensing during HCMV latency, in which viral gene expression is profoundly repressed (16, 60). Our work has identified the first inhibitor of cGAS/STING/TBK1 that is expressed and functions during latent HCMV infections within incompletely differentiated myeloid cells. In addition to inhibiting the cGAS/STING/TBK1 pathway, UL138 suppresses transcription from the viral major immediate early promoter (34, 36) that drives productive infection, and reduces the generation of infectious progeny virions during latency (27, 28). Furthermore, UL138 promotes cell surface expression of the tumor necrosis factor alpha (TNF-α) receptor (61, 62), which, when activated by an external signal, promotes many of the same pathways (e.g., NF-κB) activated by innate immunity. It would seem beneficial for the virus to maintain (or even promote) external signaling pathways leading to NF-κB activation (to support reactivation) while inhibiting internal pathogen sensing pathways that lead to NF-κB activation (to maintain latency). Potential mechanistic links between the multiple functions of UL138 and other potential innate immunity inhibitors expressed during latency remain to be explored.

## MATERIALS AND METHODS

### Cells and viruses.

Primary normal human dermal fibroblasts (NHDFs; Clonetics) and HEK293T (293T; ATCC) cells were maintained in Dulbecco’s modified Eagle medium (DMEM; Sigma) supplemented with 10% fetal bovine serum (FBS; Sigma) and 100 U/ml penicillin, 0.1 mg/ml streptomycin, and 2 mM l-glutamine (PSG; Sigma). THP-1 monocytes (ATCC) were maintained in RPMI 1640 medium (Thermo Scientific) supplemented with 10% FBS and PSG at between 2 × 10^5^ and 10 × 10^5^ cells/ml. Primary human bone marrow-derived CD34^+^ cells were purchased from StemCell Technologies (70002) and cultured in StemSpan SFEM II medium (09605; StemCell Technologies) supplemented with 1× StemSpan CC110 (containing recombinant human Flt3L, SCF, and TPO) (02697; StemCell Technologies) according to the manufacturer’s recommendations. All cells were maintained at 37°C in a 5% CO_2_ atmosphere.

HCMV wild-type AD169 and a derivative of AD169 expressing C-terminally HA-tagged UL138 under the control of its native putative promoter and polyadenylation sequences inserted between UL130 and UL131 (AD169-UL138-HA) have been previously described (34). Derivatives of TB40/E expressing enhanced GFP (eGFP) under the control of the simian virus 40 (SV40) promoter (TB40/E-GFP) and either a C-terminally tandem FLAG-tagged UL138 (TB40/E-GFP-UL138-FLAG) or an M16stop point mutant of UL138 (TB40/E-GFP-M16stop) have been previously described (35, 36). Derivatives of TB40/E-GFP encoding a C-terminally HA-tagged WT UL138 or a C-terminally HA-tagged M16stop point mutant were generated via two-step red recombination (63, 64) by first deleting the UL138 open reading frame and reinserting either wild-type UL138-HA or UL138-HA-M16stop (ATG to TAG). Recombination was performed as previously described (34, 36, 63, 64), utilizing gene blocks synthesized by Integrated DNA Technologies. Recombinant bacterial artificial chromosomes (BACs) were verified by restriction digest and Sanger sequencing. All viruses were derived from transfection of BAC clones into NHDFs, and viral stocks were concentrated by ultracentrifugation through a 20% sorbitol cushion and titers were determined by plaque assay on NHDFs. All viral stocks used had been passaged 3 times or fewer. For infection, cells were incubated with virus in minimal volume for 1 h at 37°C and then returned to normal culture volumes and incubated at 37°C for the indicated amount of time. Multiplicities of infection (MOIs) were calculated based on infectivity on NHDFs.

### Inhibitors, antibodies, and expression constructs.

Lactacystin (5 μM; Millipore Sigma), concanamycin A (50 nM; Millipore Sigma), and MG132 (5 μM; Millipore Sigma) dissolved in dimethyl sulfoxide (DMSO) were added 18 h prior to harvesting cells. Primary and secondary antibodies used in this work are listed in Table 1.

**TABLE 1:** Antibodies used in this study^*a*^

Antibody	Source or reference	Catalog no.	Use(s)
Calnexin (clone C5C9)	Cell Signaling Technology	2679	WB
cGAS (clone D1D3G)	Cell Signaling Technology	15102	WB
FLAG (clone M2)	Sigma	F3165	WB, IF, IP
GAPDH (clone 6C5)	Ambion	AM4300	WB
GM130 (clone 35)	BD Biosciences	610822	IF
GM130 (clone 6DB1)	Cell Signaling Technology	12480	WB
HA (clone 3F10)	Roche	11867431001	IF
HA (clone 16B12)	Biolegend	901502	WB
HCMV IE1 (clone 1B12)	65	NA	WB
HCMV pp71 (clone 2H10-9)	66	NA	WB
Histone H3	Abcam	ab1791	WB
IRF3 (clone FL-425)	Santa Cruz Biotechnology	sc-9082	WB
Phospho-IRF3 (Ser396) (clone 4D4G)	Cell Signaling Technology	4947	WB
Myc (clone 9E10)	Santa Cruz Biotechnology	sc-40	WB, IP
Myc tag (clone 71D10)	Cell Signaling Technology	2278	IF
Normal mouse IgG	Sigma	12-371	IP
Normal rabbit IgG	Sigma	12-370	IP
p65/RelA (clone D14E12)	Cell Signaling Technology	8242	IF
STING (clone EPR13130)	Abcam	ab181125	IF
STING (clone D2P2F)	Cell Signaling Technology	13647	WB, IP
Tubulin (clone DM1A)	Sigma	T9026	WB
IRDye680RD goat anti-mouse IgG secondary antibody	LICOR	925-68070	WB
IRDye800CW goat anti-rabbit IgG secondary antibody	LICOR	925-32211	WB
Alexa Fluor 594 goat anti-mouse IgG secondary antibody	Invitrogen	A-11005	IF
Alexa Fluor 594 goat anti-rat IgG secondary antibody	Invitrogen	A-11007	IF
Alexa Fluor 488 goat anti-rabbit IgG secondary antibody	Invitrogen	A-11008	IF
Alexa Fluor 488 goat anti-mouse IgG secondary antibody	Invitrogen	A-11001	IF
Alexa Fluor 405 goat anti-rabbit IgG secondary antibody	Invitrogen	A-31556	IF

a WB, Western blotting; IF, immunofluorescence; IP, immunoprecipitation; NA, not applicable.

Expression constructs for cGAS and STING were gifts from Blossom Damania (UNC—Chapel Hill). Expression constructs for human TBK1 and constitutively active IRF3 (IRF3-5D) were gifts from Michaela Gack (Florida Research and Innovation Center). The expression construct for NF-κB p65/RelA was a gift from Shigeki Miyamoto (University of Wisconsin—Madison). Expression constructs for mutant alleles of STING were constructed by PCR-mediated site-directed mutagenesis. The expression constructs for C-terminally HA-tagged WT UL138 and Golgi-sorting motif mutants have been previously described (34, 36, 37). Expression constructs for UL138-HA, UL138-Δ40–154, UL138-FLAG, and UL138-M16Stop were constructed by PCR amplification using wild-type or appropriate recombinant TB40/E viral DNA (34–36) as a template and subsequent cloning into pSG5 (Stratagene) using an In-Fusion HD cloning kit (638910; TaKaRa) according to the manufacturer’s instructions.

### Transfections and reporter assays.

For transfection of 293T cells, cells were seeded at 1 × 10^5^/cm^2^ in complete DMEM overnight. The medium was then changed to DMEM without antibiotics, and cells were transfected with 260 ng total DNA per 1 × 10^5^ cells using Lipofectamine 2000 (Invitrogen) at a 3:1 Lipofectamine/DNA ratio in Opti-MEM I serum-free medium (Invitrogen) according to the manufacturer’s protocol. For transfection of NHDFs, cells were seeded at 2.1 × 10^4^/cm^2^ in complete DMEM overnight. The medium was then changed to DMEM without antibiotics, and cells were transfected with 1 μg total DNA per 1 × 10^5^ cells using TransIT-X2 (Mirus) at a 2:1 TransIT/DNA ratio in Opti-MEM I serum-free medium according to the manufacturer’s protocol. For transfection of THP-1 monocytes, cells were seeded at 8 × 10^5^/ml overnight and transfected with 2.5 μg DNA per 1 × 10^6^ cells using Lipofectamine 2000 at a 3:1 Lipofectamine/DNA ratio in Opti-MEM I serum-free medium according to the manufacturer’s protocol. In all cases, medium was changed at 24 h posttransfection and cells were harvested at 48 h posttransfection.

For luciferase reporter assays, cells were cotransfected with an IFN-β promoter-driven firefly luciferase construct (a gift from Blossom Damania, UNC—Chapel Hill) along with pRL-tK or pRL-null internal *Renilla* luciferase control construct (Promega) and expression constructs for the desired proteins or a matched empty vector control. Forty-eight hours posttransfection, cells were washed with phosphate-buffered saline (PBS; Invitrogen) and lysed in 1× passive lysis buffer (PLB; Promega). Equal amounts of lysate were assayed in technical duplicate using a dual-luciferase assay (E1960; Promega) according to the manufacturer’s protocol. Luciferase activity was measured with a Veritas microplate luminometer (Turner Biosystems), firefly luciferase activity was normalized to the internal *Renilla* luciferase control, and fold induction of stimulated samples relative to unstimulated controls was calculated.

### Western blotting and immunoprecipitation.

For Western blot analysis, equal numbers of cells were washed with 1× PBS (Invitrogen) and lysed in SDS lysis buffer (1% SDS, 2% β-mercaptoethanol) or 1× passive lysis buffer (Promega). Equivalent amounts of total lysate were boiled in SDS-PAGE sample loading buffer (50 mM Tris [pH 8.0], 2% SDS, 10% glycerol, 0.0005% bromophenol blue, 6% β-mercaptoethanol), separated by SDS-PAGE, and transferred to Optitran membranes (GE Healthcare). Membranes were blocked with 5% bovine serum albumin (BSA) in TBST (10 mM Tris [pH 8.0], 150 mM NaCl, 0.05% Tween 20), incubated with primary antibody diluted in blocking buffer, washed 3 times with TBST, incubated with appropriate IRDye-conjugated secondary antibodies diluted in blocking buffer, washed again 3 times with TBST, and imaged and quantitated on an Odyssey Fc imager using Image Studio v2.1.10 software (LI-COR).

For immunoprecipitation (IP) assays, cells were lysed in IP lysis buffer (20 mM Tris-HCl [pH 7.5], 150 mM NaCl, 1 mM Na_2_EDTA, 1 mM EGTA, 1% Triton X-100, 2.5 mM sodium pyrophosphate, 1 mM beta-glycerophosphate, 1 mM Na_3_VO_4_, 1 μg/ml leupeptin, and 1 mM phenylmethylsulfonyl fluoride [PMSF]) on ice, sonicated briefly, and clarified by centrifugation. Lysates were incubated with specific antibody or an equal amount of matched IgG control antibody overnight at 4°C. Antibody complexes were isolated using protein A+G magnetic beads (88802; Thermo), and beads were washed 5 times with IP lysis buffer prior to elution of samples from the beads by boiling in SDS sample loading buffer.

### Indirect immunofluorescence.

For indirect immunofluorescence, cells were grown on glass coverslips and transfected or infected as indicated. Cells were washed in PBS, fixed with 1% paraformaldehyde, and permeabilized with PBST (PBS plus 0.1% Triton X-100 and 0.05% Tween 20). They were then blocked with 0.5% BSA and 5% goat serum (Thermo) in PBST prior to incubation with primary antibody diluted in blocking buffer. Coverslips were then washed 3 times with PBST and incubated with appropriate secondary antibodies conjugated with Alexa fluorophores (Invitrogen). Coverslips were subsequently washed 3 times with PBST, and nuclei were counterstained with Hoechst 33342 prior to being mounted on slides with Fluoromount-G (00-4958-02; Invitrogen) and imaged using either a Nikon confocal laser scanning microscope (Fig. 3A and Fig. 4A), an Olympus FluoView FV1000 confocal microscope (Fig. 1A and B and Fig. 3A), or a Leica Stellaris confocal microscope (Fig. 4E and Fig. 8A). Images were processed using Fiji software (67).

### RNA isolation and RT-qPCR.

For transcript analysis, total RNA was isolated from cells using an IBI total RNA minikit (IB145323; IBI Scientific) according to the manufacturer’s directions. Equal amounts of total RNA were treated with dsDNase and converted to cDNA using the Maxima H Minus Supermix with dsDNase system (M1682; Thermo Scientific) according to the manufacturer’s instructions. Reverse transcription-quantitative PCR (RT-qPCR) was performed using iTaq SYBR green Supermix (172-5124; Bio-Rad) and run on an ABI 7900HT real-time PCR system with SDS2.4 software (Applied Biosystems) using primers specific for IFNB1 (5′AGC AGT CTG CAC CTG AAA AGA and 5′GAC TAT GGT CCA GGC ACA GT), CXCL10 (5′AGC AGA GGA ACC TCC AGT CT and 5′ATG CAG GTA CAG CGT ACA GT), STING (5′AGC CTT GGT TCT GCT GAG TG and 5′GTA CCT GGA GTG GAT GTG GC), and glyceraldehyde-3-phosphate dehydrogenase (GAPDH) (5′GAG CCA AAA GGG TCA TC and 5′GTG GTC ATG AGT CCT TC). Each biological replicate was measured in technical triplicate, and expression levels were normalized to GAPDH and calculated relative to wild-type virus-infected cells from the same experiment using the comparative threshold cycle (*C_T_*) method (68).

### Data presentation and statistics.

All bar graphs show the means ± standard errors of the means (SEM) from the indicated number of independent biological replicates. All blots and micrographs shown are representative images from the indicated number of independent biological replicates. Statistical significance was determined by two-tailed Student’s *t* test and in figures is represented as follows: *, *P* < 0.05; **, *P* < 0.01; ***, *P* < 0.001; and ns, *P* > 0.1.
